# From Chronic Inflammation to Remodeling: Anthocyanins in the Context of Asthma Management

**DOI:** 10.3390/ph19020323

**Published:** 2026-02-15

**Authors:** Madiha Ajaz, Indu Singh, Lada Vugic, Rati Jani, Ayesha Zahid, Natalie Shilton

**Affiliations:** 1 School of Pharmacy and Medical Sciences, Griffith University, Gold Coast QLD 4222, Australia; i.singh@griffith.edu.au (I.S.); l.vugic@griffith.edu.au (L.V.); a.zahid@griffith.edu.au (A.Z.); n.shilton@griffith.edu.au (N.S.); 2 School of Health Sciences and Social Work, Griffith University, Gold Coast QLD 4222, Australia; r.jani@griffith.edu.au; 3 Institute for Biomedicine and Glycomics, Griffith University, Gold Coast QLD 4222, Australia

**Keywords:** airway remodeling, anthocyanins, asthma, inflammation, antioxidants

## Abstract

Asthma is a prevalent chronic disease posing substantial health and economic challenges globally. Its progression involves key hallmarks such as inflammation and airway remodeling, mediated by multiple inflammatory biomarkers and pathways. Despite the availability of potent therapeutic options, many patients continue to suffer from uncontrolled asthma. The plasminogen activator inhibitor-1 (PAI-1) signaling pathway is critical in asthma exacerbation and remodeling, with elevated PAI-1 levels linked to disease progression. Anthocyanins (ACNs), potent antioxidants and anti-inflammatory compounds, have shown promise in asthma management. Epidemiological studies associate higher ACN intake with a lower risk of asthma and improved lung function. Preclinical models further demonstrate ACNs’ effectiveness in reducing asthma-related inflammatory cytokines, chemokines, and signaling pathways. Additionally, a human trial suggests ACNs can improve symptom control and lung function. While no direct evidence links ACNs to PAI-1 reduction in asthma, studies in other chronic conditions show ACNs reduce PAI-1 levels, supporting their potential role in asthma. This suggests a promising avenue for exploring their effects on airway remodeling. The lack of robust human studies remains a gap. Future research should focus on establishing direct evidence of ACNs’ impact on PAI-1 levels and remodeling in asthma, providing novel insights into managing asthma as an adjunct.

## 1. Introduction

Asthma is a chronic inflammatory disease of the airways characterized by symptoms such as cough, wheezing, dyspnea, and breathing difficulties. These manifestations arise from airway obstruction caused by hyperresponsiveness and inflammation. As a non-communicable condition, asthma can be life-threatening if inadequately managed [1,2]. It imposes a substantial global health and economic burden, with approximately 300 million people affected worldwide and 5.8 mortalities per 100,000 individuals yearly. The associated healthcare costs and productivity losses further exacerbate this burden [3,4].

The pathophysiology of asthma is multifactorial, with diverse etiological drivers and a heterogeneous presentation [5,6]. Current pharmacological treatments, including corticosteroids, beta-agonists, biologics, and leukotriene receptor antagonists, are generally effective; however, a significant proportion of patients continue to experience uncontrolled asthma despite optimal therapy [7,8]. Persistent inflammation in such cases is closely linked to airway remodelling, a phenomenon characterized by structural and functional alterations in the airways that exacerbate disease severity and progression [9,10].

Emerging evidence highlights the role of the plasminogen activator inhibitor-1 (*PAI-1*) gene in asthma pathogenesis. Elevated PAI-1 levels are linked with fibrin deposition, contributing to airway remodelling and inflammation [11,12,13,14,15]. Targeting PAI-1 presents a promising therapeutic avenue, with studies suggesting its inhibition may reduce asthma exacerbations, mitigate inflammation, and limit extracellular matrix deposition [16,17]. Moreover, inhaled corticosteroid (ICS) use at high doses has been shown to promote a profibrinogenic environment, further underscoring the need for alternative or adjunctive strategies to manage asthma and its complications [18,19].

Given certain limitations of conventional treatments, including high costs and occasional side effects, integrative interventions have gained attention as complementary strategies to enhance treatment outcomes. Interventions such as massage, exercise, and herbal or nutritional therapies have demonstrated potential benefits [20,21,22,23,24,25,26,27,28,29]. For instance, prolonged adherence to a predominantly plant-based diet has been associated with slower lung function decline, while antioxidant-rich compounds like flavonoids exhibit anti-inflammatory effects in asthma [30,31].

Anthocyanins, a subclass of flavonoids, have demonstrated efficacy in reducing inflammation and exhibiting nutraceutical effects across various metabolic conditions [32,33,34,35,36]. However, their potential to mitigate asthma-related inflammation and PAI-1 levels remains unexplored. This review examines anthocyanins’ potential role in addressing airway inflammation and remodelling in asthma.

## 2. Brief Overview of Anthocyanins

**Figure 1:**
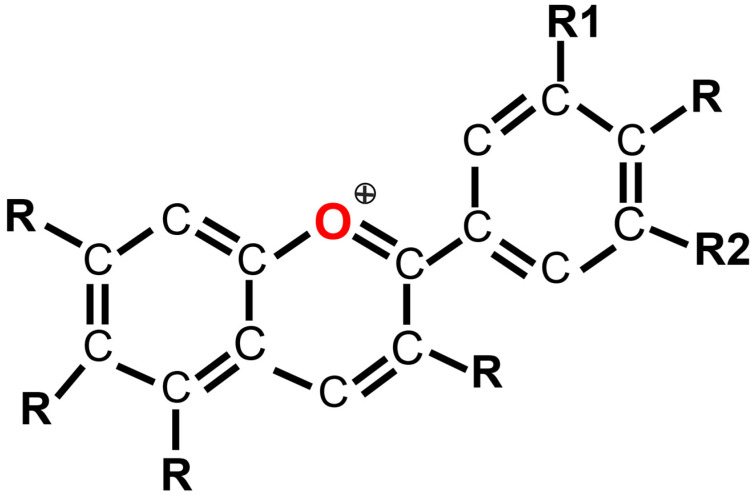
Basic Chemical Structure of Anthocyanin. Abbreviation: +, chromenylium (flavylium) cation. Created with BioGDP.com, GDP2026YJTH2M.

Anthocyanins (ACNs) are a subclass of polyphenols, representing important phytochemicals, flavonoids [37]. The basic flavone structure of the anthocyanins consists of a three-carbon bridge connecting two aromatic rings (Figure 1). While anthocyanidins are in aglycone (non-sugar) forms, anthocyanins are in their glycosylated forms, having varying numbers of hydroxyl and methoxy groups, and different sugar moieties, which makes them more stable than anthocyanidins [38,39]. Conjugated bonds in anthocyanins are responsible for the pigmentation in plant tubers, flowers, and fruits, producing red, purple, or blue colors. There are more than 650 types of anthocyanins available from plants. Cyanidin, delphinidin, malvidin, pelargonidin, peonidin, and petunidin are the most typical anthocyanidins, and they serve as the main constituents of 90% of the anthocyanins found in nature [37]. Berries, red wine, red to purple-colored fruits, vegetables, and edible flowers are all rich sources of anthocyanins [37]. Extracted anthocyanins are natural colorants with no side effects, but anthocyanins’ stability is sensitive to processing techniques, type of pigment, heat, light, oxygen, enzyme activity, co-pigments, and metal ions [37,40]. However, advanced techniques and measures such as encapsulation methods for drug delivery, co-pigmentation, compatible metal ion usage, and utilizing protein-binding techniques to enhance food matrices have been utilized to improve stability during digestion and bioavailability [41,42,43,44,45]. Jejunum and ileum have been reported as the primary absorption sites for glycosylated anthocyanins. ACNs interact with the gut microbiota in a bidirectional manner, which contributes to their biological effects. On one hand, ACNs can influence the composition and function of the gut microbial community, partly through selective stimulation or inhibition of specific bacterial taxa. On the other hand, gut microbes metabolize ACNs into various low–molecular-weight phenolic compounds, including cyanidin and phenolic acids, which are the key absorbed metabolites. It is plausible that these metabolites account for the biological activities of anthocyanins [46,47], considering that both anthocyanins and phenolic acids exhibit similar biological activities [48]. Table 1 summarizes dietary sources, major anthocyanin compounds, and the bioavailability of anthocyanins [49].

## 3. Antioxidant Activities of Anthocyanins

**Figure 2:**
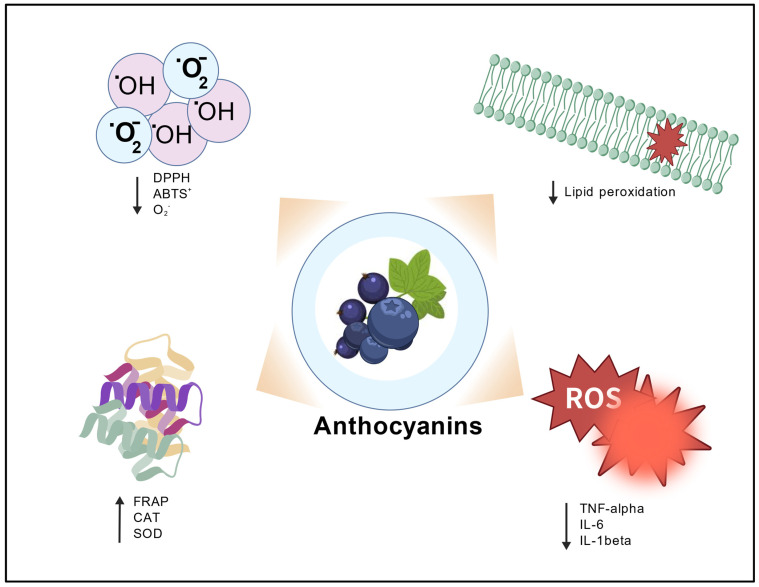
Schematic representation of the antioxidant actions of anthocyanins, illustrating their role in neutralizing free radicals, reducing oxidative damage, enhancing antioxidant enzyme activity, and protecting against oxidative stress and inflammation. Created with BioGDP.com, GDP2026IJTHMA. Abbreviations: ↑, increase; ↓, decrease; DPPH, 2,2-Diphenyl-1-picrylhydrazyl; ABTS^+^, 2,2′-Azino-bis (3-ethylbenzothiazoline-6-sulfonic acid) radical cation; FRAP, Ferric Reducing Antioxidant Power; SOD, Superoxide Dismutase; CAT, Catalase; TNF-alpha, Tumour Necrosis Factor-alpha; IL, Interleukin; O_2_^−^, Superoxide anion radical.

There is credible evidence for anthocyanins’ antioxidant [51,52,53,54,55], anti-inflammatory [56,57,58], and health-promoting biological activities [59,60,61,62]. Increased reactive oxygen species production and reduced antioxidant defence systems of the body result in oxidative stress, damaging cells and organs, and stimulating inflammation [63,64,65]. Due to an intrinsic lack of electrons, anthocyanins are naturally reactive and potent antioxidants. Their unique chemical structure facilitates the neutralization of free radicals [66,67]. Multiple studies highlight the antioxidant potential of anthocyanins. Blueberry anthocyanins’ capacity to neutralize free radicals, including ABTS^+^, DPPH, and O_2_^−^, was determined by Li et al. They also found that anthocyanins boost FRAP and improve reducing power [68]. In addition, anthocyanins extracted from blueberries enhanced the activity of catalase (CAT) and total superoxide dismutase (T-SOD) [69]. Similarly, Li et al. tested the antioxidant activity of anthocyanin monomers prepared from black chokeberry in mice with renal ischemia, which enhanced the activity of superoxide dismutase (SOD), CAT, and glutathione (GSH), while suppressing the production of tumour necrosis factor-α (TNF-α), interleukin-6 (IL-6), interleukin-1β (IL-1β), monocyte chemoattractant protein-1 (MCP-1), and malondialdehyde (MDA) [70]. Coklar and Akbulut found that anthocyanins extracted from *Mahonia aquifolium,* berries demonstrated twice the antioxidant activity of other phenolic fractions extracted from the same berries [71]. Moreover, Ogawa et al. evaluated the potential of *Vaccinium myrtillus* L. anthocyanidins as an antioxidant. The findings revealed significant concentration-dependent free radical (OH, O_2_^−^) scavenging activity and reduction in lipid peroxidation [72] (Figure 2). These studies suggest the potential of anthocyanins as antioxidants; however, uncertainty regarding the molecular mechanism warrants further research.

## 4. Anti-Inflammatory Activities of Anthocyanins

**Figure 3:**
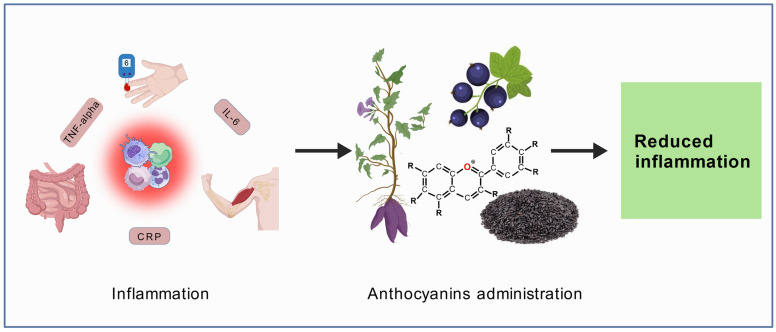
Schematic representation of the anti-inflammatory effects of anthocyanins, highlighting their role in reducing key inflammatory markers such as IL-6, CRP, and TNF-α, and their potential impact on chronic conditions, including diabetes, obesity, skeletal muscle inflammation, and colitis. Abbreviations: →, proposed sequence; +, chromenylium (flavylium) cation. Created with BioGDP.com, GDP2025KJT7MA.

Inflammation is derived from inflammare, a Latin word, referring to ignition or burning which manifests as swelling, pain, redness, dysfunction, and heat. Acute inflammation is harmless when it responds to infections or tissue insults, etc., and terminates after eliminating the damage. Several mediators are involved in the immune processes for resolving inflammation. However, failure to resolve inflammation due to the continuous presence or spread of inflammatory triggers, like oxidative stress, microorganisms, etc., leads to chronic inflammation [73,74,75]. There is growing evidence of the anti-inflammatory potential of anthocyanins. Mu et al., Chen et al., and Li et al. reported similar findings, demonstrating the anti-inflammatory efficacy of anthocyanins extracted from purple sweet potato and black rice in attenuating Dextran Sodium Sulphate (DSS)-induced colitis in a murine model [76,77,78]. Black currant extract, rich in anthocyanins, has been shown to reduce inflammation and increase plasma levels of IL-10 when supplemented daily for 5 weeks pre- and two hours post-30 min rowing sessions [79]. Anthocyanin supplementation (320 mg/day for 4 weeks) extracted from bilberries reduced IL-6, IL-18, and TNF-α levels in individuals at risk of type 2 diabetes [80]. Likewise, the obesity-linked pro-inflammatory biomarker IL-6 was attenuated by a 28-day supplementation with anthocyanin capsules (320 mg/day) derived from blackcurrant and bilberries [81]. Several systematic reviews and meta-analyses documented the anti-inflammatory response of anthocyanin supplementation [82,83,84]. Markers of vascular and systemic inflammation including C-reactive protein (CRP), IL-6, TNF-α, intercellular adhesion molecule-1, and vascular adhesion molecule-1 (VCAM-1) were significantly reduced after ACN intake in a dose-dependent manner [85]. Another systematic review and meta-analysis identified that ACN supplementation significantly decreases TNF-α. A non-significant decrease was also observed in IL-6 and high-sensitivity C-reactive protein (hs-CRP) levels [86]. Additionally, Hariri et al. conducted a systematic review and meta-analysis to assess the impact of purified ACN supplementation on inflammatory mediators, including serum levels of CRP, TNF-α, and IL-6. The findings indicate a significant reduction in serum CRP, TNF-α, and IL-6 levels [34]. These findings support the anti-inflammatory potential of ACNs and highlight their potential use as a nutraceutical in various inflammatory conditions (Figure 3).

## 5. Role of Anthocyanins in Asthma

Asthma is a chronic condition with diverse clinical presentations and treatment responses, exhibiting a multifaceted progression throughout an individual’s lifetime [87]. Environmental pollutants, allergens, exercise, or occupational irritants can trigger allergic asthma, primarily associated with type 2 high (Th2-high) inflammation. Th2-high asthma, which accounts for over 50% of cases, is driven by eosinophilic activity and Th2 lymphocytes. These cells release cytokines such as interleukin-4 (IL-4), IL-5, and IL-13, leading to mucus hypersecretion, airway obstruction, and an increase in smooth muscle mass. In contrast, non-allergic asthma, often associated with type 2 low (Th2-low) or non-Th2 mechanisms, remains poorly understood in terms of its underlying pathological drivers. Th2-low asthma is driven by Th1 and Th17 pathways and is characterized by elevated levels of pro-inflammatory cytokines, including tumor necrosis factor-alpha (TNF-α), IL-1β, IL-8, IL-6, and IL-17A. This phenotype also features neutrophil infiltration, excessive mucus production, and structural airway changes that lead to remodelling [87,88]. Additionally, anti-inflammatory cytokines such as IL-10, IL-35, and IL-37 play regulatory roles. A decreased ratio of interferon-gamma (IFN-γ) to IL-4 has also been associated with asthma progression [89,90,91,92,93].

Chemokines from the CC and CXC families, such as CCL-1, 2, 3, 17, 22, RANTES, eotaxin, and CX3CL1, along with cytokines like thymic stromal lymphopoietin (TSLP) and IL-33, play a crucial role in airway inflammation and asthma development. These mediators are secreted by airway epithelial cells in response to triggers such as microbes, cellular stress, or immunoglobulin E (IgE) mediated inflammation, amplifying the inflammatory response [94,95,96,97,98,99].

Complex signaling pathways are closely associated with inflammation, hyperresponsiveness, and structural alterations of the airways in asthma. The Janus Kinase-Signal Transducer and Activator of Transcription (JAK-STAT) axis, activated by IL-4 and IL-13, is fundamental to Th2 asthma, promoting IgE production and mucus secretion. Antigen recognition through pathogen- or damage-associated molecular patterns activates the toll-like receptor (TLR) pathway, which subsequently triggers nuclear factor-kappa B (NF-κB), interferon regulatory factor (IRF), and mitogen-activated protein kinase (MAPK) pathways, critical mediators of inflammatory cytokine and chemokine release, as well as immune responses. Functionally, p38 MAPK and JNK (c-Jun N-terminal kinase) pathways respond to inflammatory stimuli, translating signals into cellular responses that include the transcription and translation of pro-inflammatory genes. By activating transcription factors, they drive the synthesis of inflammatory mediators, which in turn can create a positive feedback loop that exacerbates inflammation. Nuclear factor erythroid 2-related factor 2 (NRF2), under chronic inflammatory conditions, induces transforming growth factor-beta (TGF-β) release, contributing to fibrosis and inflammation. Additionally, an imbalance in the Foxp3-RORγt (Treg/Th17) axis plays a role in asthma-linked immune dysregulation and inflammation severity. Calcium signalling pathways drive smooth muscle contraction, leading to airway hyperresponsiveness and bronchoconstriction, while chemoattractant receptor-homologous molecule expressed on Th2 cells (CRTH2) facilitates eosinophil stimulation and migration, key processes in airway inflammation. In asthma pathogenesis P13K has a critical role, involved in regulating immune cell activation, inflammation, and airway remodeling. Inhibiting the PI3Kβ and mTOR pathways influences the immune response and defense against pathogens by affecting crucial cellular functions like immune cell activation, metabolism, and cytokine production. The PI3K-mTOR pathway is essential for cell growth and survival, but when dysregulated (for example, by pathogens), it can lead to immune dysregulation. Targeting this pathway with inhibitors can potentially modulate these effects, for example, by affecting T-cells. In conjunction with other contributing mechanisms, these pathways shape the complex pathophysiology of asthma, presenting critical avenues to target inflammation, oxidative stress, and structural changes [100,101,102,103].

Epidemiological studies have demonstrated the bioactive properties of anthocyanidins and anthocyanins, with expected outcomes including reduced asthma risk and improved health markers associated with increased intake [104,105,106,107,108]. However, both Garcia-Larsen et al. and Wu et al. relied on dietary surveys with self-reported flavonoid intake instead of direct measurements, with limited control for confounders, limiting causal inference between flavonoid intake and chronic respiratory outcomes. Moreover, one case–control study found no significant association between anthocyanin intake and asthma risk, possibly due to the stable nature of asthma in the included population, which typically exhibited reduced inflammation or the uneven sample distribution of five cases to one control (Table 2) [109].

Evidence from multiple in vitro studies supports the anti-inflammatory and anti-asthmatic potential of ACNs through the regulation of key inflammatory markers and pathways. ACNs have been shown to suppress the levels of CCL11 (eotaxin-1), CCL26 (eotaxin-3), and NF-κB activation [111,112,113], which are pivotal mediators of airway hyperreactivity and eosinophilic inflammation in asthma and core markers of airway remodeling [114,115]. Additionally, ACNs effectively downregulated histamine, caspase-1, TSLP, and pro-inflammatory cytokines, including IL-6, TNF-α, and IL-1β [116]. These pro-inflammatory cytokines function as acute-phase responders, while the alarmin cytokine TSLP plays a central role in allergen-induced asthma pathophysiology. Targeting TSLP has been shown to mitigate bronchoconstriction, inflammation, and airway hyperresponsiveness [117,118]. Moreover, caspase-1, a key enzyme in the pyroptosis pathway, is critical for the activation of IL-1β and IL-18 precursors [119] (Table 3).

Although animal model studies are limited still, they highlight the role of ACNs and structurally related compounds in asthma. Cyanidin is a typical anthocyanin. It significantly downregulated the expression of asthma-linked pro-inflammatory cytokines, including IL-4, IL-5, IL-13, and GATA-binding protein 3 (GATA3) expression. Cyanidin was also effective in modulating IL-4Rα, total signal transducer and activator of transcription 6 (STAT6), p-STAT6, miR-138-5p, total JAK1, and p-JAK1expressions and reducing percent eosinophil and neutrophil, the eosinophil/lymphocyte ratio, and mucus production in the asthma mouse model [120,122]. In addition, anthocyanins presented potent anti-inflammatory effects by reducing the infiltration of T cells, eosinophils, and neutrophils, accompanied by inhibiting mucus production and upregulating Arginase-1 (Arg1), Chitinase-3-like protein 3 (Ym-1), and Fizz1 [121]. Arg1, Ym-1, and Fizz-1 play significant roles in asthma severity and promote structural changes in the airways associated with collagen deposition and airway fibrosis [123,124,125]. Anthocyanins also enhanced the production of CXCL10 and CCL4 chemokines, which the authors considered as asthma modulation effects in the context of their study, referring to their role in attracting macrophages/monocytes at inflammatory sites and reducing infiltration of eosinophils [126] (Table 2). However, it is important to exercise caution when interpreting these findings, as the doses used in animal models (often expressed in mg/kg) are typically much higher than what can be achieved through normal dietary intake. Moreover, animal models do not perfectly replicate human physiology, so these results cannot be directly extrapolated to humans. Therefore, these findings should not be generalized to imply that consuming anthocyanin-rich foods alone can prevent or treat asthma.

Trials evaluating the impact of anthocyanins on asthma outcomes in humans are scarce. Watson et al. conducted a randomized trial to analyze the effectiveness of passion fruit peel extract (150 mg/day), a rich source of cyanidin-3-O-glucoside, quercetin-3-O-glucoside, and edulilic acid, on asthma management in adults. The supplementation led to a significant improvement in asthma symptoms, including cough, wheezing, and shortness of breath, as well as in forced vital capacity (FVC). However, forced expiratory volume in one second (FEV1) did not show any improvement [110,121] (Table 3). The aqueous extract of *viola odorata* flowers is rich in anthocyanins, flavonoids, coumarin, salicylic acid, saponins, and other bioactive compounds. In a placebo-controlled study, violet syrup was administered three times daily at a dosage of 2.5 cc for children aged 2–5 years and 5 cc for those aged 5 years and older. The treatment resulted in an age-dependent, significant reduction in cough among children with intermittent asthma without any side effects. Notably, the study did not specifically assess the effects of anthocyanins [127]. Furthermore, a study that identified and evaluated the antioxidant potential of Cordyline terminalis purple flowers, traditionally used to treat asthma, revealed the presence of five anthocyanins and significant antioxidant activity, supporting their potential use as an anti-asthma remedy [128].

Overall, preclinical and human evidence suggest that ACNs and proanthocyanidins are most effective in Type-2 (Th2-high) asthma, particularly allergic and eosinophilic phenotypes and chronic asthma with airway remodeling. Their consistent suppression of Th2 cytokines (IL-4, IL-5, IL-13), eosinophilic chemokines, eosinophil recruitment, and mucus production supports relevance for allergic and eosinophilic asthma. In chronic models, ACNs/PACs also reduce airway remodeling markers (e.g., TGF-β1, α-SMA, collagen, fibrosis), indicating potential benefit in long-standing disease. Some extracts additionally enhance M2 macrophage–associated anti-inflammatory responses. However, currently, no evidence supports efficacy in other asthma phenotypes. These studies can be viewed as preliminary evidence suggesting anthocyanins’ potential effectiveness in alleviating asthma-related inflammation and symptoms. Considering the supporting preclinical evidence, further investigation into the effects of anthocyanins in humans is warranted.

## 6. Anthocyanins’ Potential in Modulating PAI-1 Levels in Asthma

**Figure 4:**
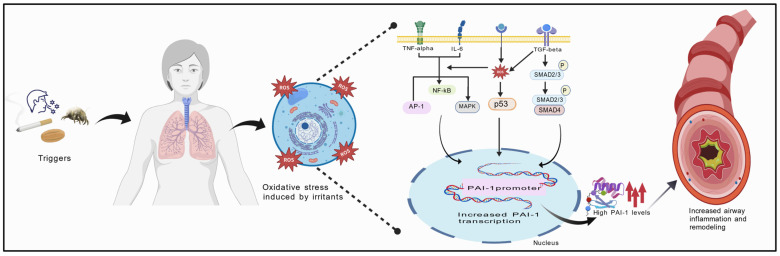
Schematic Representation of Inflammation and Remodeling in Asthma. Oxidative stress, resulting from exposure to irritants, leads to the production of inflammatory cytokines and chemokines, along with the activation of inflammatory pathways. The cascade of inflammatory responses upregulates PAI-1 expression, contributing to fibrosis, airway remodeling, and chronic inflammation. Abbreviations: Arrows represent sequence; dotted lines represent proposed intracellular mechanism. Created with BioGDP.com, GDP2025IJT7M0.

Structural remodeling is one of the critical pathological aspects of asthma, marked by alterations in the cellular and extracellular matrix (ECM) of the airways alongside epithelial cell death, fibroblast stimulation, and the growth of airway smooth muscle cells [129]. Collagen fibers, elastin, glycosaminoglycans (GAGs), proteoglycans, adhesion protein fibronectin, etc., constitute the extracellular matrix, with collagen being the predominant component [129,130]. Irritants stimulate fibroblasts, airway smooth muscle, and epithelial cells to produce ECM proteins [131,132,133,134]. Plasminogen Activator Inhibitor-1 (PAI-1) is a key mediator of fibrosis within the superfamily of serine protease inhibitors. It drives the buildup of the extracellular matrix (ECM) by inhibiting tissue-type plasminogen activator (t-PA) and urokinase-type plasminogen activator (u-PA) [100,135], which are responsible for fibrin polymer lysis and the conversion of plasminogen to plasmin. Beyond its role in fibrosis, PAI-1 also contributes to inflammation, as in the *PAI-1* gene’s promoter region, a conserved distal NF-_K_B binding site, has been recognized [136] (Figure 4).

Literature suggests that PAI-1 levels are elevated in asthma [15,137,138]. Conversely, the absence or inhibition of *PAI-1* have been associated with reductions in inflammation, airway remodelling, and hyperresponsiveness [139,140]. A genetic polymorphism, specifically the 4G/5G variation in the transcriptional control region of the *PAI-1* gene, has been shown to influence plasma PAI-1 levels, with the 4G allele being linked to higher expression [141]. Furthermore, a meta-analysis provides evidence that the 4G allele is significantly associated with an increased risk of asthma, particularly in adults, identifying the −675 4G/5G polymorphism as a potential genetic risk factor for asthma susceptibility [14]. Inhaled corticosteroids, though potent in asthma management, have been associated with a pro-fibrinogenic state, especially in high doses or severe asthma cases [18,19].

As per our knowledge, ACNs have not yet been studied for their ability to modulate PAI-1 levels specifically in asthma. However, evidence from other contexts suggests their potential. For instance, proanthocyanidins are distinct polymeric flavonoids that hydrolyze in acidic conditions and release anthocyanidins [142]. Grape seed extract, which is rich in proanthocyanidins, was administered intraperitoneally in a murine model of chronic asthma induced by ovalbumin (OVA). After eight weeks, the treatment reduced inflammatory markers such as IL-4, IL-13, VEGF, eosinophils, and IgE. It also attenuated airway remodeling, as indicated by decreased collagen deposition and lower TGF-β1 expression [115]. It is important to note that TGF-β1 contributes to fibrosis by stimulating the transcription of *PAI-1* [143,144].

The anti-aging gene Sirtuin-1 (*SIRT1*) has a significant role in the regulation of airway inflammation. Reduced *SIRT1* activity has been linked to impaired pulmonary function and elevation of key pro-inflammatory cytokine levels, including IL-4, IL-5, and IL-13 [145,146,147]. In addition, it has also been identified as a negative regulator of *PAI-1,* suppressing its transcription through epigenetic mechanisms [148]. Interestingly, ACNs have been identified to upregulate *SIRT1* levels and alleviate inflammation in in vivo and in vitro models of asthma [122].

Although ACNs have not been directly investigated in relation to PAI-1 regulation in asthma, evidence from other experimental models indicates several biologically plausible mechanisms. ACNs are known to modulate key inflammatory and profibrotic signalling pathways, including inhibition of NF-kB, AP-1 and MAPK cascades, regulation of p53 activity, and suppression of TGF-β–mediated Smad2/3–Smad4 signalling linked with pulmonary fibrosis, all of which contribute to transcriptional control of PAI-1. These pathways are closely linked to airway inflammation, remodelling, and fibrotic responses in asthma, processes in which PAI-1 plays a pivotal role [149,150,151,152,153,154].

Beyond transcriptional regulation, PAI-1 expression is subject to post-transcriptional control by microRNAs such as miR-30 and the miR-143/145 cluster, which destabilise PAI-1 mRNA [155,156]. While direct evidence is lacking, ACN-mediated anti-inflammatory and redox-modulating effects may indirectly influence these microRNA networks [157,158]. Taken together, these mechanistic insights provide a coherent rationale for a potential modulatory role of ACNs on PAI-1 in asthma, warranting targeted investigation in this disease context.

**Figure 5:**
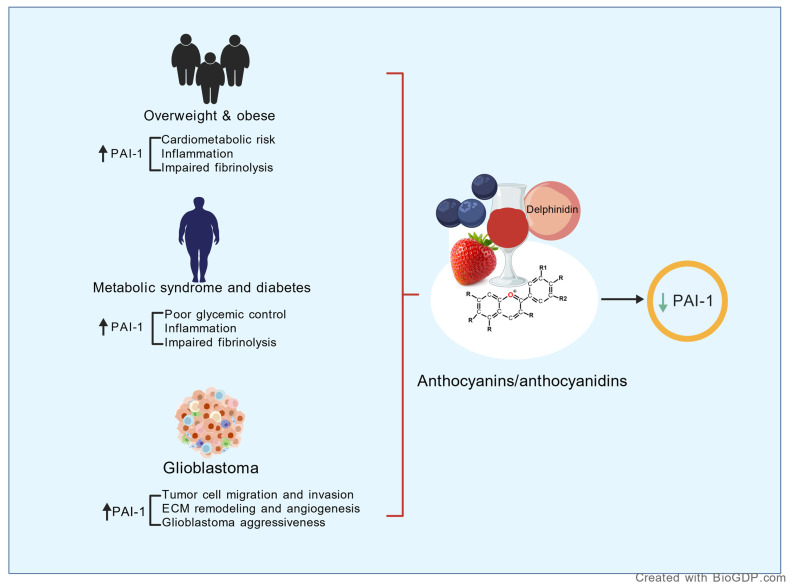
Impact of anthocyanin intake on reducing PAI-1 levels across chronic inflammatory conditions, including cancer, diabetes, and obesity. Abbreviations: ↑, high levels; ↓, low levels; +, chromenylium (flavylium) cation; }, groups conditions with high PAI-1 levels; →, proposed effect. Created with BioGDP.com, GDP20265JAFAF.

In cancer research, ACNs, including cyanidin, delphinidin, and petunidin, have been shown to influence plasminogen activation. Delphinidin, in particular, was observed to reduce *PAI-1* expression while enhancing u-PA levels [159]. Furthermore, a meta-analysis highlighted that ACN supplementation significantly decreased PAI-1 levels, improving glucose metabolism and glycemic control, especially in individuals with diabetes [33]. Similarly, a randomized controlled trial demonstrated that a strawberry drink rich in ACNs and polyphenols reduced postprandial PAI-1 levels in overweight participants [160] (Figure 5). On the other hand, studies involving hypercholesterolemic patients showed no significant decrease in PAI-1 levels with ACN supplementation, though a minor reduction was reported. Researchers proposed that increasing the intervention duration or dose might yield stronger effects [161].

While the efficacy of ACNs in modulating PAI-1 levels in asthma remains unstudied, evidence from other conditions suggests a possible interaction. However, this link is speculative, and the observed benefits of ACNs in airway remodeling may arise from broader anti-inflammatory and antioxidant mechanisms rather than PAI-1-specific effects. Further research is needed to clarify whether PAI-1 modulation contributes meaningfully to these outcomes.

Also, despite emerging evidence, several translational challenges need consideration before therapeutic application of anthocyanins. Like many polyphenols, anthocyanins exhibit dose-dependent and sometimes biphasic effects, complicating effective and safe dose selection for human use [162,163]. In whole foods, anthocyanins are embedded within complex nutrient and polyphenol matrices that shape absorption, metabolism, and synergistic interactions, meaning that purified supplements may not replicate the physiological benefits of anthocyanin-rich foods. Emerging data suggest that whole-food sources may offer superior bioavailability, though this remains to be systematically validated. Furthermore, the intrinsic low stability, rapid metabolism, and poor bioavailability of anthocyanins reduce the predictability of their biological effects, while inter-individual differences in gut microbiota–dependent metabolism lead to heterogeneous responses [164]. Collectively, these challenges underscore the need for standardized formulations, novel delivery strategies, and rigorously designed human trials to enable the reliable translation of anthocyanins into therapeutic applications.

This review paper is extended version of abstract published elsewhere [152].

## 7. Conclusions

PAI-1 has a crucial impact on airway remodeling and inflammation in asthma, with its inhibition linked to reduced fibrosis and improved lung function. While no studies have directly examined ACNs’ effects on PAI-1 in asthma, evidence from epidemiological, bench, and clinical investigations suggests their anti-inflammatory and anti-asthmatic potential. Given that ACNs have been shown to reduce PAI-1 levels in other chronic conditions, additional investigations are needed to explore their role in airway remodeling and asthma management as adjuncts.

## Figures and Tables

**Table 1:** Major Anthocyanin compounds’ dietary sources and bioavailability.

Anthocyanidin	Dietary Sources	Major Anthocyanins	Chemical Structure		Bioavailability
			R1	R2	
Pelargonidin	Strawberry, red leaf	Pelargonidin-3-O-galactoside, Pelargonidin-3-O-glucoside, Pelargonidin-3-O-arabinoside	H	H	Low levels of intact anthocyanins (<1%). Higher systemic availability when metabolites are included. Bioavailability is influenced by structure (glycosylation, acylation) and gut microbiota metabolism [50].
Cyanidin	Blackberry/black rice, black beans, dark berries	Cyanidin-3-O-galactoside, Cyanidin-3-O-glucoside, Cyanidin-3-O-arabinoside	OH	H	
Delphinidin	Berries (blueberry/bilberry-type), red cabbage	Delphinidin-3-O-galactoside, Delphinidin-3-O-arabinoside, Delphinidin-3-O-glucoside	OH	OH	
Peonidin	Purple carrots, berries, and vegetables	Peonidin-3-O-galactoside, Peonidin-3-O-arabinoside, Peonidin-3-O-acetyl-galactoside	OCH_3_	H	
Petunidin	Eggplant, berries	Petunidin-3-O-galactoside, Petunidin-3-O-glucoside, Petunidin-3-O-arabinoside	OH	OCH_3_	
Malvidin	Grapes, blueberries, wine grapes	Malvidin-3-O-glucoside, Malvidin-3-O-arabinoside	OCH_3_	OCH_3_	

Abbreviations: OH, hydroxyl group; OCH_3_, methyl ether group

**Table 2:** Summary of human studies evaluating the impact of anthocyanin intake on asthma outcomes.

Author, Year	Study Design	Sample, Age	Exposure/Intervention	Participants’ Clinical Condition	Results
Larsen et al., 2018 [104]	Cross-sectional	2599, 15–74 years old.	Dietary total flavonoids and proanthocyanidins (measured using FFQ).	Asthma (self-reported), chronic sinusitis, both, or none.	53% less likelihood of FVC < LLN, ↑ FEV_1_/FVC with ↑proanthocyanidins and flavonoid intake.
Wu et al., 2024 [105]	Cross-sectional	15,753, 18 years or more.	Dietary anthocyanidins intake measured by 24 h recalls.	2193 survey participants had asthma.	↓ asthma prevalence with ↑intake of anthocyanidins.
Suna et al., 2023 [106]	Cross-sectional	60, 19–50 years old.	Dietary intake measured by semi-quantitative FFQ	30 people with asthma, 30 controls	↓ intake of anthocyanidins among people with asthma, and ↑intake of anthocyanidins among controls.
Mattioli et al., 2020 [109]	Case–control	990, 20–84 years old.	Dietary intake measured by FFQ (with a focus on total flavonoids and flavanones, anthocyanins, flavan-3-ols, flavonols, flavones, polymers, and proanthocyanidins	237 survey participants had stable asthma	NS association between anthocyanins or anthocyanidins intake with asthma
Watson et al., 2008 [110]	Randomized placebo-controlled trial	43, 18–60 years old	Passion fruit peel extract 150 mg/day (cyanidin-3-O-glucoside, quercetin-3-O-glucoside, and edulilic acid)	Patients with asthma (22 in the intervention, while 21 in the control group)	↓ cough, wheeze, shortness of breath ↑ FVC NS, FEV_1_
Bondonno et al., 2024 [107]	Cohort study	119,466, 40–69 years old.	Dietary intake measured by a 24 h dietary recall questionnaire (with a focus on flavonols, flavones, flavan-3-ol monomers, proanthocyanidin s, flavanones, theaflavins + thearubigins, and anthocyanins).	14,388 people with asthma.	NS association between total flavonoids and asthma risk. ↑ flavonols, flavones, flavan-3-ol monomers, proanthocyanidins, flavanones, and anthocyanins intake was associated with ↑ percent predicted FEV_1_
Sun and Ding, 2024 [108]	Cross-sectional	11,743, ≥20 years old.	Flavonoid intake was assessed using 24- hour dietary recall (for two days) and WWIE methods (with a focus on Daidzein, Petunidin, Eisenstein, Glycitein, Pelargonidin, Cyanidin, Delphinidin, Malvidin, Peonidin, Catechin, Epigallocatechin 3-gallate, Epigallocatechin, Epicatechin, Epicatechin 3-gallate, Theaflavin, Thearubigins, Apigenin, Eriodictyol, Hesperetin, Naringenin, Luteolin, Isorhamnetin, Myricetin, Quercetin, Theaflavin-3,3′-digallate, Theaflavin-3′-gallate, Kaempferol, Theaflavin-3-gallate, and Gallocatechin)	1693 people with asthma.	↑ total flavonoid intake associated with ↓ asthma risk ↑ Peonidin, Eriodictyol, and Luteolin intake were associated with ↑ health benefits for asthma.

Abbreviations: ↑, increased; ↓, decreased; FFQ, food frequency questionnaire; FVC, forced vital capacity; LLN, lower limit of normal; FEV_1_, forced exhaled volume in 1 s.

**Table 3:** Summary of asthma-related markers modulated by anthocyanins and proanthocyanidins in preclinical studies, including changes in cytokines, chemokines, immune cell infiltration, signaling pathways, and airway remodeling indicators.

Author, Year	Study Type	Model Used	Exposure/Intervention	Asthma Stimulus/Induction	Results
Nyanhanda et al., 2014 [113]	In vitro	A549 cells	Different cultivators’ Black Currant extracts (in combinations of delphinidin glucoside and cyanidin glucoside OR delphinidin rutinoside and cyanidin rutinoside)	IL-4 (10ng/mL)	↓ CCL26 levels
Shaw et al., 2017 [112]	Animal and In vitro	Mouse model Human lung epithelial cells	Anthocyanins rich black currant extract (delphinidin and cyanidin)	OVA	↓ levels of CCL11, eosinophils and inflammation
Jeon et al., 2019 [116]	In vitro	HMC-1	*Schisandra chinensis* extract (Cyanidin 3-Rutinoside)	PMA/A23187	↓ IL-6, TNF-α, IL-1β, histamine, caspase-1, TSLP levels
Peng et al., 2020 [111]	In vitro	A549 cells	Anthocyanin rich purple Kiwi fruit extract	TNF-α (5ng/mL) and IL-4 (5ng/mL)	Modulated CCL11secretion and NF-_K_B activation
Zhou et al., 2015 [115]	Animal	32 BALB/c mice (female), 8-week-old	Intraperitoneally injected grape seed extract (GSPE) in doses of 50 mg·kg^−1^·d^−1^	OVA	↓ AHR, BALF total cells & eosinophils, peribronchiolar/perivascular inflammation, PAS-positive goblet cells (mucus), peri bronchial fibrosis & collagen, lung hydroxyproline, α-SMA, TGF-β1, BALF IL-4, IL-13, VEGF; serum total IgE.
Ma et al., 2019 [120]	Animal	BALB/c mice (4 weeks old)	Cyanidin-3-O-β-glucoside (extracted from black rice skin)	OVA	↓ percent eosinophils, neutrophils, ratio of eosinophil/lymphocyte, mucus production, BALF levels of IL-4, IL-5, IL-13, and mRNA expression of IL-4, IL-5, GATA3, expression of IL-4Rα, total STAT6, p-STAT6, total Jak1, and p-Jak1 NS: BALF levels of IFN-γ, IL-17A, IL-10, mRNA expression of IFN-γ, IL-13 T-bet, RORc, and Foxp3
Shaw et al., 2021 [121]	Animal	C57BL/6J mice.	Apple and boysenberry juice concentrate with total anthocyanins 2.5 mg/kg.	OVA challenge	↓ mucous production, and count of eosinophils, neutrophils, and T cells. ↑ gene expression of arginase (Arg1), chitinase 3-like 3 (Ym-1), and Fizz1 ↑ chemokine production (CXCL10 and CCL4)
Liu et al., 2022 [122]	In vitro and animal model	70 BALB/c mice (7–8 weeks old) Human bronchial epithelial cells (16HBE14o-160)	Standard anthocyanin extract (cyanidin-3-O glucoside)	OVA	↓ IL-4, IL-5, IL-13 levels, downregulated miR-138-5p expression ↑ IFN-γ levels

Abbreviations: ↑, increased; ↓, decreased; IL, interleukin; CCL, C-C motif chemokine ligand; HMC-1, human mast cell line-1; TNF-α tumor necrosis factor alpha; TSLP, thymic stromal lymphopoietin; NF-κB, nuclear factor-kappa B; IgE, immunoglobulin E; AHR, airway hyperresponsiveness; BALF, bronchoalveolar lavage fluid; GATA3, GATA binding protein 3; STAT6, signal transducer and activator of transcription 6; Jak1 janus kinase 1; IFN-γ, interferon gamma; T-bet, T-box transcription factor; RORc, retinoic acid-related orphan receptor gamma; Foxp3, forkhead box protein P3; A549, human alveolar basal epithelial cell line.

## Data Availability

No new data were created or analyzed in this study. Data sharing is not applicable to this article.

## Abbreviations

The following abbreviations are used in this manuscript:

ACNs	Anthocyanins
PAI-1	Plasminogen activator inhibitor-1
ICS	Inhaled corticosteroid
